# Chemical Inhibition of Histone Deacetylases 1 and 2 Induces Fetal Hemoglobin through Activation of GATA2

**DOI:** 10.1371/journal.pone.0153767

**Published:** 2016-04-13

**Authors:** Jeffrey R. Shearstone, Olga Golonzhka, Apurva Chonkar, David Tamang, John H. van Duzer, Simon S. Jones, Matthew B. Jarpe

**Affiliations:** Acetylon Pharmaceuticals Inc., Boston, MA, United States of America; Schulze Center for Novel Therapeutics, Mayo Clinic, UNITED STATES

## Abstract

Therapeutic intervention aimed at reactivation of fetal hemoglobin protein (HbF) is a promising approach for ameliorating sickle cell disease (SCD) and β-thalassemia. Previous studies showed genetic knockdown of histone deacetylase (HDAC) 1 or 2 is sufficient to induce HbF. Here we show that ACY-957, a selective chemical inhibitor of HDAC1 and 2 (HDAC1/2), elicits a dose and time dependent induction of γ-globin mRNA (*HBG*) and HbF in cultured primary cells derived from healthy individuals and sickle cell patients. Gene expression profiling of erythroid progenitors treated with ACY-957 identified global changes in gene expression that were significantly enriched in genes previously shown to be affected by *HDAC1* or *2* knockdown. These genes included *GATA2*, which was induced greater than 3-fold. Lentiviral overexpression of GATA2 in primary erythroid progenitors increased *HBG*, and reduced adult β-globin mRNA (*HBB*). Furthermore, knockdown of *GATA2* attenuated *HBG* induction by ACY-957. Chromatin immunoprecipitation and sequencing (ChIP-Seq) of primary erythroid progenitors demonstrated that HDAC1 and 2 occupancy was highly correlated throughout the *GATA2* locus and that HDAC1/2 inhibition led to elevated histone acetylation at well-known *GATA2* autoregulatory regions. The GATA2 protein itself also showed increased binding at these regions in response to ACY-957 treatment. These data show that chemical inhibition of HDAC1/2 induces *HBG* and suggest that this effect is mediated, at least in part, by histone acetylation-induced activation of the *GATA2* gene.

## Introduction

SCD and β-thalassemia are recessive hemoglobinopathies that specifically affect the β-globin subunit of adult hemoglobin. They are among the most common genetic disorders in the world [1]. In SCD, a single amino acid substitution leads to formation of hemoglobin with lowered solubility when deoxygenated (HbS) [2]. HbS aggregation leads to distortion of the red blood cell into a rigid, sickle-like shape. These cells undergo premature lysis, resulting in chronic anemia, and block small blood vessels, reducing oxygen delivery to tissues. In β-thalassemia, a diverse panel of genetic changes leads to reduced or absent expression of the adult β-globin gene [3]. Ineffective erythropoiesis results from unpaired α-globin chains which form precipitates that lead to oxidative damage and lysis of red blood cell precursors. The chemotherapeutic agent hydroxyurea is currently the only drug with proven efficacy in SCD [4, 5]. However, this therapy only reduces the frequency and severity of sickle cell crises in a subset of patients. There are no approved therapies for β-thalassemia beyond transfusion and bone marrow transplantation.

The β-globin locus is arranged as a cluster of five functionally similar β-like globin genes that are sequentially activated, then deactivated, at discrete developmental stages [6]. This process is referred to as globin switching. For example, following birth, *HBG* is repressed and replaced with *HBB* expression, which persists throughout adult life. The HBG protein is able to pair with adult α-globin to yield a fetal hemoglobin molecule, HbF, whose presence inhibits aggregation of HbS in SCD and reduces excess α-globin in β-thalassemia [2, 3]. Consistent with this observation, the severity of SCD and β-thalassemia is reduced in individuals that retain elevated levels of HbF as adults [7, 8]. Moreover, induction of HbF by hydroxyurea in SCD patients is directly proportional to improved clinical response [4, 5]. Therefore, therapeutic interventions aimed at *HBG* induction are a promising approach for ameliorating SCD and β-thalassemia.

Regulation of β-like globin gene expression is mediated by variety of epigenetic and chromatin-modifying factors. For example, elevated *HBG* expression is observed following genetic or chemical inhibition of DNA methylation [9], the methyl-cytosine binding protein MBD2 [10], the histone arginine methyltrasferase PRMT5 [11], the histone lysine demethylase KDM1A [12, 13], the histone methyltransferases EHMT1 and EHMT2 [14, 15], and the zinc-dependent histone deacetylases (HDACs), a group of enzymes that remove acetyl groups, primarily from histone lysines. Inhibition of HDAC activity results in elevated histone acetylation which has been associated with increased chromatin accessibility and gene expression. A variety of non-selective HDAC inhibitors have been used successfully to induce HbF in preclinical studies [16–18]. However, the use of non-selective HDAC inhibitors in the clinical setting has been associated with significant toxicity and adverse events [19, 20]. Recent reports have shown that genetic knockdown of *HDAC1* or *HDAC2* individually, but not *HDAC3*, is sufficient to induce HbF in adult erythroid progenitor cells [21–23]. Therefore, development of selective and potent HDAC1/2 inhibitors leading to HbF induction represents a refined and targeted therapeutic approach for the treatment of SCD and β-thalassemia. In this work, we have used a novel HDAC1/2-selective small molecule, ACY-957, to investigate the role of HDAC1/2 in the regulation of the *HBG* gene.

## Materials and Methods

### Cell culture

CD34+ cells isolated from human bone marrow of healthy normal donors (Lonza, AllCells, or StemCell Technologies), referred to as BM cells throughout this work, were cultured using two distinct 2-phase culture systems. These systems have been described previously and are referred to as Culture System 1 (CS1) [24] and Culture System 2 (CS2) [21] throughout this work. CS1 expansion media is StemSpan SFEM (StemCell Technologies) supplemented with CC100 (StemCell Technologies), 100 U/mL penicillin and 100 μg/mL streptomycin. CC100 is a mix of Flt-3 ligand (FLT3LG), KIT ligand (KITLG), interleukin-3 (IL3), and interleukin-6 (IL6). CS1 differentiation media is StemSpan SFEM supplemented with 1 U/ml erythropoietin (EPO), 5 ng/mL IL3, 20 ng/mL KITLG, 2 μM dexamethasone, 1 μM beta-estradiol, 100 U/mL penicillin and 100 μg/mL streptomycin. CS2 expansion media is StemSpan SFEM supplemented with 1% glutamine, 40 μg/mL lipids (Sigma), 100 ng/mL KITLG, 10 ng/mL IL3, and 0.5 U/mL EPO. CS2 differentiation media is same as CS2 expansion media, except 3 U/mL EPO.

Peripheral blood from patients homozygous for the sickle cell mutation (Conversant Bio) were collected in heparinized tubes and shipped overnight on cool packs. Patients had not had a transfusion for at least 6 months prior to sample collection. Peripheral blood mononuclear cells (PBMC) were isolated using a Ficoll-Paque Plus (GE Healthcare Lifesciences) density gradient in SepMate tubes (StemCell Technologies). PBMCs were cultured in CS1 expansion media for 7 days. Cells lacking GYPA expression were isolated using the Glycophorin A Depletion Kit (StemCell Technologies) and placed in differentiation media for 3 to 5 days in the presence of drug or vehicle control. Alternatively, PBMCs were cultured in CS1 expansion media for 3 days, and then hematopoietic progenitor cells were selected using the EasySep Human Progenitor Cell Enrichment Kit (StemCell Technologies). Progenitor cells were placed back into CS1 expansion media for an additional 4 days, and then shifted to differentiation media for 3 days in the presence of drug or vehicle control. Differentiation media for this experimental series has been described previously [25] and is composed of Iscove's Modified Dulbecco's Medium supplemented with 10 ng/mL KITLG, 3 U/mL EPO, 2% human peripheral blood plasma, 3% human AB serum, 200 μg/mL holo-transferrin, 3 IU/mL heparin, 10 μg/mL insulin, 100 U/mL penicillin and 100 μg/mL streptomycin.

Burst forming unit erythroid (BFU-E) assays were performed by ReachBio Research Labs (Seattle, WA). On the day of the experiment, normal human bone marrow mononuclear cells (Lonza) were thawed rapidly, diluted in 10 mL of IMDM containing 10% fetal bovine serum and washed by centrifugation. The supernatant was discarded and the cell pellet resuspended in a known volume of IMDM + 10% FBS. ACY-957 or vehicle was added to a semi-solid methylcellulose-based media formulation containing IL3 (10 ng/mL), CSF2 (10 ng/mL), KITLG (50 ng/mL), and EPO (3 U/mL) and plated in triplicate. BFU-E assays were initiated at 2.5 x 10^4^ cells per culture. Following 14–16 days in culture, BFU-E colonies were isolated and RNA purified using the RNeasy Micro Kit (Qiagen).

### HDAC enzyme assays

*In vitro* biochemical assays were performed as described previously [26]. Specifically, drugs were dissolved and diluted in assay buffer (50 mM HEPES, pH 7.4, 100 mM KCl, 0.001% Tween-20, 0.05% BSA, and 20 μM Tris (2-carboxyethyl)phosphine) to 6-fold the final concentration. HDAC enzymes (BPS Biosciences) were diluted to 1.5-fold of the final concentration in assay buffer and pre-incubated with ACY-957 for 24 hours before the addition of the substrate. The amount of substrate (acetyl-lysine tripeptide substrate for HDAC1, HDAC2, HDAC3, and HDAC6 or trifluoroacetyl-lysine tripeptide for HDAC4, HDAC5, HDAC7, HDAC8, and HDAC9) used for each enzyme was equal to the Michaelis constant (Km), as determined by a titration curve. Substrate was diluted in assay buffer to 6-fold the final concentration with 0.3 μM sequencing grade trypsin (Sigma-Aldrich). The substrate/trypsin mix was added to the enzyme/compound mix and the plate was shaken for 5 seconds and then placed into a SpectraMax M5 microtiter plate reader (Molecular Devices). The enzymatic reaction was monitored over 30 minutes for release of 7-amino-4-methoxy-coumarin after deacetylation of the lysine side chain in the peptide substrate, and the linear rate of the reaction was calculated.

The HDAC-Glo 2 Kit (Promega) was used for *in vivo* HDAC2 inhibition assays [27, 28]. First, BM cells were expanded in CS1 for 6 days. Next, 25000 cells in 25 μL of CS1 expansion media was placed in a 384-well plate in the presence of compound. Cells were cultured for an additional 48 hours, followed by luminescence detection in the lytic format as described by the manufacturer’s protocol.

### Western blots

The EpiQuick Total Histone Extraction kit (Epigentek) was used to isolate histones. Histone lysates were size separated on Bolt 4–12% Bis-Tris gradient gels (Life Technologies), transferred to polyvinylidene difluoride membrane and detected using standard western blotting technique. GATA2 protein was detected by lysing cells in RIPA denaturing buffer supplemented with protease inhibitors followed by detection using a Wes capillary electrophoresis system (Protein Simple) or western blotting on nitrocellulose membrane. Antibodies used for western blotting: Anti-GATA2 [clone EPR2822(2)] (Abcam), anti-ACTB [clone 1E35] (Cell Signaling Technology), anti-H3K9/14 [catalog number 17–615] (EMD Millipore), anti-H3K56ac [clone EPR996Y] (Abcam), anti-H3K79 [catalog number 07–750] (EMD Millipore), anti-H2BK5 [catalog number 2574] (Cell Signaling Technology), anti-H3K9me3 [catalog number 07–442] (EMD Millipore), and anti-total-H4 [clone 62-141-13] (EMD Millipore).

### β-like globin mRNA detection

Globin mRNA was detected using quantitative real time PCR (QPCR). Total RNA was isolated using RNeasy Micro Kit (Qiagen) with on-column DNase digestion, converted into cDNA using the High Capacity RNA-to-cDNA Kit (Life Technologies), diluted 5-fold with water, and 1 μL used as a template for QPCR. The percent of each β-like globin transcript was determined using the absolute standard curve method. Briefly, DNA corresponding to the full length transcript for each globin isoform was cloned into pU57 plasmid (GenScript, Piscataway NJ). Each plasmid was validated against each QPCR probe to ensure specificity. Plasmids were pooled at equal molar concentration and a dilution series spanning 8-logs was utilized as the standard curve. Throughout this work, ‘*HBG* mRNA (%)’ and ‘percent *HBG* mRNA’ refers to the abundance of *HBG* mRNA expressed relative to the sum of the abundances of all β-like globin transcripts, i.e. [*HBG*/(*HBB*+*HBD*+*HBG*+*HBE*)]*100. In cases where the transcript of interest was compared against the housekeeping gene *ACTB*, the comparative Ct (2^ΔΔCt^) method was utilized. Probes used for QPCR: *HBB* (Hs00747223_g1), *HBD* (Hs00426283_m1), *HBG* (Hs00361131_g1, detects both *HBG1* and *HBG2*), *HBE* (Hs00362215_g1), *GATA1* (Hs01085823_m1), *GATA2* (Hs00231119_m1), *KLF1* (Hs00610592_m1), *MYB* (Hs00920556_m1), *SOX6* (Hs00264525_m1), *BCL11A* (Hs01093197_m1), and *ACTB* (Hs99999903_m1).

### Flow cytometry

Flow cytometry was performed on a FC500 flow cytometer (Becton Dickenson). Fetal hemoglobin protein was assayed using the Fetal Hemoglobin Test Kit (Life Technologies) according to manufacturer’s protocol. Samples were stained with FITC-conjugated mouse IgG1 anti-human HbF antibody (clone HbF-1, Life Technologies). Each sample was also stained with a FITC-conjugated mouse IgG1 isotype control antibody (clone X40, BD Biosciences) and used as a negative control. FETALtrol cells were used as positive controls (Trillium Diagnostics). TFRC and GYPA were detected on fresh cells by staining with 20 μL of PE-conjugated anti-human TFRC (clone M-A712, catalog 555537, BD Biosciences) and 0.5 μL of FITC- or APC-conjugated anti-human GYPA (clone GA-R2 (HIR2), catalog 559943 or 551336, BD Biosciences) in 200 μL PBS with 0.1% BSA for 40 min on ice. Forward and side scatter, or staining with 7AAD (catalog 559925, BD Biosciences), was used to identify viable cells.

### Gene expression profiling and data analysis

BM cells were cultured in CS1 expansion media for 7 days, and then shifted to CS1 differentiation media containing vehicle or 1 μM ACY-957 for 5 days. Total RNA was isolated from cells at day 5 of differentiation using RNeasy Micro Kit with on-column DNase digestion (Qiagen). This experiment was performed in triplicate using cells from three independent donors on separate days to obtain vehicle treated (n = 3) and ACY-957 treated (n = 3) samples for GeneChip analysis. GeneChip sample preparation, hybridization, and data acquisition was performed by the Covance Genomics Laboratory (Seattle, WA) using Affymetrix GeneChip PrimeView^™^ Human Gene Expression Array according to manufacturer’s protocol. This data has been uploaded to the National Center for Biotechnology Information (NCBI) Gene Expression Omnibus (GEO) website with series accession number of GSE60791 under super series accession number GSE60793. For the analysis of *HDAC1* or *HDAC2* knockdown in erythroblasts, raw gene expression data was downloaded from NCBI GEO website, series GSE22366 [21].

Data analysis was performed using BRB-ArrayTools [29]. GSE60791 and GSE22366 data series were imported and analyzed independently. Raw data CEL files were processed with the robust multi-chip average (RMA) algorithm [30]. Fold changes were derived from the geometric mean of intensity values in each treatment class. In the case of GSE60791, P-values were generated between ACY-957 and vehicle treated samples using a paired *t* test with random variance model applied. In the case of GSE22366, P-values were generated between luciferase control and *HDAC1* or *HDAC2* knockdown samples using a two-sample *t* test with random variance model applied.

For gene set enrichment analysis (GSEA), gene sets for *HDAC1* or *HDAC2* knockdown were compiled as follows. ‘Up in *HDAC1* KD’ gene set includes genes which were induced at least 1.5-fold or more by *HDAC1* KD at a P-value less than 0.025. ‘Up in *HDAC2* KD’ gene set includes genes which were induced at least 1.5-fold or more by *HDAC2* KD at a P-value less than 0.025. ‘Down in *HDAC1* KD’ gene set includes genes which were repressed at least 1.5-fold or more by *HDAC1* KD at a P-value less than 0.025. ‘Down in *HDAC2* KD’ gene set includes genes which were repressed at least 1.5-fold or more by *HDAC2* KD at a P-value less than 0.025. Duplicate gene symbols were removed by selecting the probe set with the lowest P-value. Next, these 4 gene sets were appended to the Molecular Signatures Database collection of Chemical and Genetic Perturbations. This database contained approximately 2777 gene sets, each containing at least 15 genes and no more than 10000 genes. Finally, log base 2 mRNA data from ACY-957 or vehicle treated cells was loaded into the publically available GSEA software from the Broad Institute and queried against the database of gene sets described above [31]. ACY-957 phenotype was compared against vehicle phenotype by first collapsing the data set to gene symbols based on maximum probe signal and then using a weighted, signal-to-noise metric for ranking genes. Gene set permutations were performed to generate nominal P-values for each gene set.

### Chromatin immunoprecipitation

Cells were fixed in 1% formaldehyde for 15 minutes at room temperature. Crosslinking reaction was extinguished by adding glycine to 125 mM. Cells were washed three times in PBS, 0.5% lgepal CA-630, 1 mM phenylmethanesulfonyl fluoride (PMSF). Cell pellets were snap frozen on dry ice and stored at -80°C.

ChIP-Seq was performed by Active Motif (Carlsbad, CA). Chromatin (30 μg) from vehicle (n = 1) or ACY-957 (n = 1) treated cells was immunoprecipitated (n = 1, each) with antibodies against GATA2 (20 μL, sc-9008, Santa Cruz Biotechnology), HDAC1 (20 μg, ab7028, Abcam), or HDAC2 (4 μg, ab12169, Abcam). ChIP DNA, as well as a control sample composed of equal amounts of input DNA from each treatment group, was processed into Illumina libraries using standard procedures, and libraries were sequenced on HiSeq 2000. The 50-nt sequence reads were mapped to the human genome, build Hg19, using the BWA algorithm with default settings. For the analysis, all data files were normalized to the same number of unique alignments without duplicate reads (24 million). Sequence tags were extended *in silico* (using Active Motif software) at their 3′-ends to a length of 150 bp. To identify the density of fragments (extended tags) along the genome, the genome was divided into 32-nt bins and the number of fragments in each bin was determined. The resulting histogram files (bigwig format) are presented in figures using the Santa Cruz Genome Browser. Visualizations were exported as an eps file and formatted using Adobe Illustrator. Regions of GATA2 enrichment (intervals) were determined using MACS peak calling software [32], which identifies enrichments in the ACY-957 or vehicle treated ChIP data file when compared to the input DNA data file (random background). To compare peak metrics between vehicle and ACY-957 treated samples, overlapping intervals are grouped into active regions, which are defined by the start coordinate of the most upstream interval and the end coordinate of the most downstream interval. In locations where only one sample has an interval, this interval defines the active region. The ratio of average fragment densities within an active region was used to determine the fold enrichment. All ChIP-seq data is available at the NCBI GEO under accession GSE60792. GeneChip and ChIP-seq data is grouped as GEO superseries GSE60793.

ENCODE tracks were derived from K562 cells and have been labeled with gray text. Track labels: ‘Chr state’ refers to the ‘Chromatin State Segmentation by HMM from ENCODE/Broad’ data track with GEO sample accession GSM936088, ‘DNase I’ refers to the ‘DNaseI/FAIRE/ChIP Synthesis from ENCODE/OpenChrom (Duke/UNC/UTA)’ data track with GEO sample accession GSM1002657, and ‘5C’ refers to ‘Chromatin Interactions by 5C from ENCODE/Dekker Univ. Mass’ data track with GEO sample accession GSM970500. ‘HDAC1’ and ‘HDAC2’ refer to ‘Histone Modifications by ChIP-seq from ENCODE/Broad Institute’ data track with GEO sample accession GSM1003448 and GSM1003447, respectively. ‘GATA1’ and ‘GATA2’ refers to the ‘Transcription Factor Binding Sites by ChIP-seq from ENCODE/Stanford/Yale/USC/Harvard’ data track with GEO sample accession GSM935540 and GSM935373, respectively.

For ChIP-QPCR experiments, chromatin (11 μg) from vehicle (n = 1) or ACY-957 (n = 1) treated cells was fragmented by sonication, then immunoprecipitated (n = 3, each) using antibodies against histone H3 lysine 9 acetylation (3 μg, ab10812, Abcam), histone H2B lysine 5 acetylation (6 μL, ab40886, Abcam), histone H3 lysine 27 acetylation (3 μg, ab4729, Abcam), or GATA2 (6 μg, SC-9008, Santa Cruz). Specific genomic regions within immunoprecipitated and input DNA was detected using SYBR green QPCR with the following primer pairs:

*GATA2*–1.8kb; Fw CACACCATCCAGACCTTCCT, Rv CCTCCCACAGACAGACAGAA

*GATA2*–2.8kb; Fw CGTGTTTGGATGGACACG, Rv CTCGCACACCTGCACTTTT

*GATA2*–3.9kb; Fw GCAGCCATTTTCCCTATCTC, Rv TCGCAGGTTGTGTGACTTTG

*GATA2* +9.5kb; Fw CGCATTATTTGCAGAGTGGA, Rv CTCAGCTCAGTCCTGCCTCT

*INO80* (Ctrl); Fw CGGCCACTTTCACTCACTG, Rv ATTGTTCTGGCCTGGCTATG

*HBD* 1; Fw TCCCTTAACTTGCCCTGAGA, Rv GCGGTGGGGAGATATGTAGA

*HBD* 2; Fw ATATCTCCCCACCGCATCTC, Rv TTAGCCTAAAACACTTCTGC

*HBB*; Fw CTCAGGAGTCAGATGCACCA, Rv AGTCAGGGCAGAGCCATCTA

### Overexpression and shRNA constructs

shRNA experiments were conducted using the RNAi consortium (TRC) TRC1.5 vector, also known as pLKO.1-puro (Sigma). Five vectors containing shRNA targeting *GATA2* were screened in expanded BM cells and the two hairpins yielding the greatest knockdown of *GATA2* mRNA were selected for further use: shG2-1, TRCN0000019264 and shG2-2, TRCN0000019265. The non-mammalian shRNA control (Sigma, SHC002) does not target any mouse or human genes and was used as a control (shCtrl). Lentiviral supernatants were produced in the 293T cell line using shRNA vectors, viral packaging mix (Sigma, SHP001), and TransIT-293 transfection reagent (Mirus Bio, Madison, WI). BM cells were grown for 3 days in CS1 expansion media, and then transduced using lentiviral supernatant’s supplemented with 5 μg/mL of polybrene under room temperature centrifugation at 1200 x g for 75 minutes. Transduced cells were grown for two days to allow for expression of construct, followed by selection for two days in 1 μg/mL of puromycin. The *GATA2* overexpression vector (oeG2) was constructed by removing the GFP cassette of the pLKO.1-puro-CMV-TurboGFP vector (Sigma, SHC003) at the 5' NheI and 3' PstI cloning sites and replacing it with the full-length *GATA2* transcript sequence (Refseq NM_001145661) (GenScript, Piscataway, NJ). pLKO.1-puro-CMV-TurboGFP vector was used as a control (oeGFP). BM cells were grown for 3 days in CS1 expansion media, and then transduced using the RetroNectin-bound virus method according to manufacturer’s protocol. Briefly, 35 mm RetroNectin pre-coated cell culture plates (Clontech) were incubated with 2 mL of viral supernatant for 5 hours at 33ᵒC. Supernatant was removed and 5 x 10^5^ cells in 2.5 mL of CS1 expansion media was added. Transduced cells were grown for two days to allow for expression of construct, followed by selection for two days in 1 μg/mL of puromycin. Cells were then cultured for an additional 4 days in CS1 differentiation media.

## Results

### ACY-957 is an HDAC1/2-selective inhibitor

**Fig 1:**
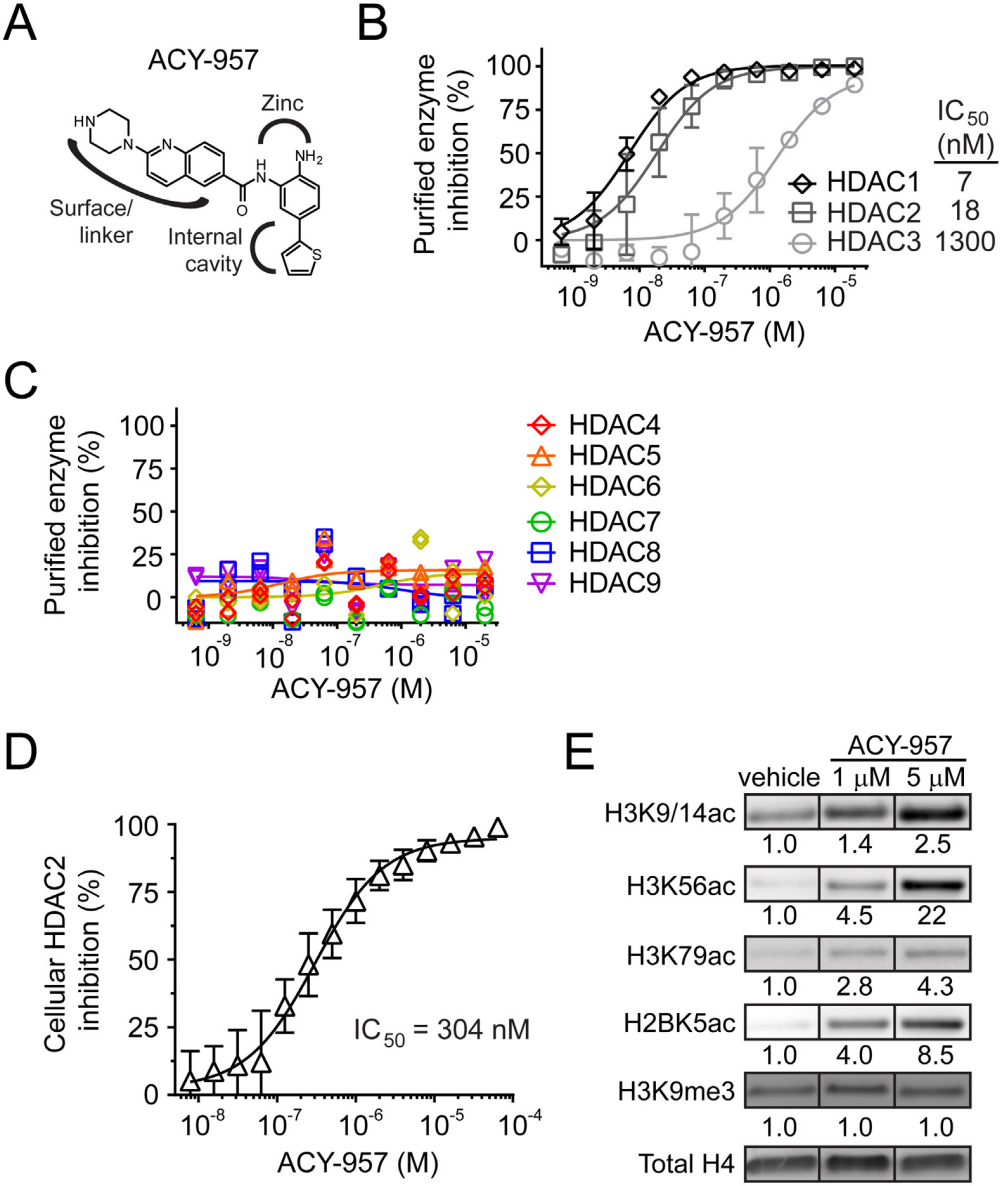
ACY-957 is an HDAC1/2-selective inhibitor. (A) Chemical structure of the HDAC1/2-selective inhibitor ACY-957. The HDAC active site consists of a tubular pocket and a zinc binding domain. The benzamide scaffold contains a linker/surface region that binds the tubular pocket and a region which coordinates zinc. The internal cavity binding group provides selectivity for HDAC1/2. (B) *In vitro* biochemical assay for HDAC1, HDAC2, or HDAC3 inhibition by ACY-957. Assay was repeated on separate days with duplicates for each concentration (mean ± SD, n = 4). (C) *In vitro* biochemical assay for HDAC4, 5, 6, 7, 8, and 9 inhibition by ACY-957. (D) Cellular HDAC2 inhibition. BM cells were cultured in CS1 expansion media for 6 days followed by treatment with ACY-957 for an additional 48 hours. Assay was performed in triplicate for each of two different donor cells cultured on different days (mean ± SD, n = 6). (E) Dose-dependent induction of histone acetylation by ACY-957. BM cells were cultured in CS2 expansion media for 6 days followed by treatment with drug for an additional 24 hours and then examined by western blot. Values represent relative abundance of each acetylation or methylation mark relative to total histone H4 and then normalized to vehicle control.

The HDAC active site consists of a tubular pocket and a zinc binding domain. The aminobenzamide class of HDAC inhibitors contain a surface/linker region that binds the tubular pocket and a zinc-coordinating aminobenzamide group. These compounds inhibit HDAC1/2/3, but display no activity against any other HDAC [26]. MS-275 (entinostat) is the most clinically advanced compound in this class [33]. Extending this work, biaryl aminobenzamides with an internal cavity binding region were shown to provide selectivity exclusively towards HDAC1/2 [34, 35]. We synthesized a library of these HDAC1/2-selective compounds, from which ACY-957 (Fig 1A) was chosen for further study based on its HDAC selectivity profile, described in more detail below, and for its favorable pharmacokinetic properties in mouse, rat, dog, and monkey [36].

ACY-957 was tested against each individual HDAC in an *in vitro* biochemical assay as previously described [26]. Aminobenzamides have slow association rate constants, therefore a prolonged pre-incubation time of 24 hours is required to reach equilibrium [37]. ACY-957 had IC_50_ values of 7 nM, 18 nM, and 1300 nM against HDAC1/2/3, respectively (Fig 1B). As expected, no inhibition of HDAC4/5/6/7/8/9 by ACY-957 was observed at concentrations as high as 20 μM (Fig 1C). Therefore, ACY-957 is an HDAC1/2 inhibitor with an approximately 100-fold selectivity over HDAC3. This selectivity profile is consistent with those reported for compounds in the same biaryl aminobenzamide structural class as ACY-957 [35, 37].

Next, ACY-957’s ability to inhibit HDAC activity in primary hematopoietic progenitors derived from human bone marrow was investigated. Using an acetylated substrate selective for HDAC2 [27, 28], we found that ACY-957 had an IC_50_ value of 304 nM (Fig 1D), a value approximately 10-fold higher than obtained in the biochemical assay with recombinant HDAC2 (Fig 1B). It is not uncommon for biochemical and cell-based assays to yield different IC_50_ values for a variety of potential reasons, including the compound’s diffusion across the nuclear and cellular membranes, active efflux from the cell, impaired inhibition of HDAC2 within large multiprotein complexes present in the cellular context, or a different local concentration of competing substrate within the cell. To further validate histone deacetylase inhibition in cells, primary human erythroblasts were cultured with ACY-957 for 24 hours, and then histone acetylation was examined by western blot. Treatment with 1 μM or 5 μM ACY-957 led to a dose-dependent accumulation of acetylation on histone H3 lysine 9 and 14 (H3K9/14ac), H3 lysine 56 (H3K56), H3 lysine 79 (H3K79ac), and H2B lysine 5 (H2BK5ac), while histone H3 lysine 9 tri-methylation (H3K9me3) was not affected by ACY-957 treatment (Fig 1E). Together, these findings demonstrate that ACY-957 efficiently crosses cellular and nuclear membranes to inhibit HDAC1/2.

### Selective inhibition of HDAC1/2 by ACY-957 induces HbG mRNA and HbF protein

To evaluate the ability of ACY-957 to activate *HBG*, we utilized two distinct 2-phase culture systems, referred to as CS1 [24] and CS2 [21], to derive erythroid progenitors and erythroblasts from CD34+ human bone marrow cells (BM cells). Unless otherwise noted, throughout this work ‘day 0’ refers to the time at which cells were switched from expansion media to erythroid differentiation media. We first characterized the differentiation stage of cells in each of these systems by flow cytometry using fluorescent antibodies against the transferrin receptor (TFRC) and glycophorin A (GYPA) [38]. CS1 cells were TFRC^neg^GYPA^neg^ following the expansion phase (day 0), maturing to TFRC^pos^GYPA^mid^ after 5 days in differentiation media. In contrast, CS2 cells were TFRC^pos^GYPA^neg/mid^ following expansion phase, maturing to TFRC^pos^GYPA^pos^ after 5 days in differentiation media. Therefore, in the experiments described below, CS1 media contains cells at the earliest stages of erythroid maturation, including BFU-E, CFU-E, and proerythroblasts, while CS2 media contains cells at slightly later stages of erythroid maturation, including proerythroblasts and basophilic erythroblasts.

**Fig 2:**
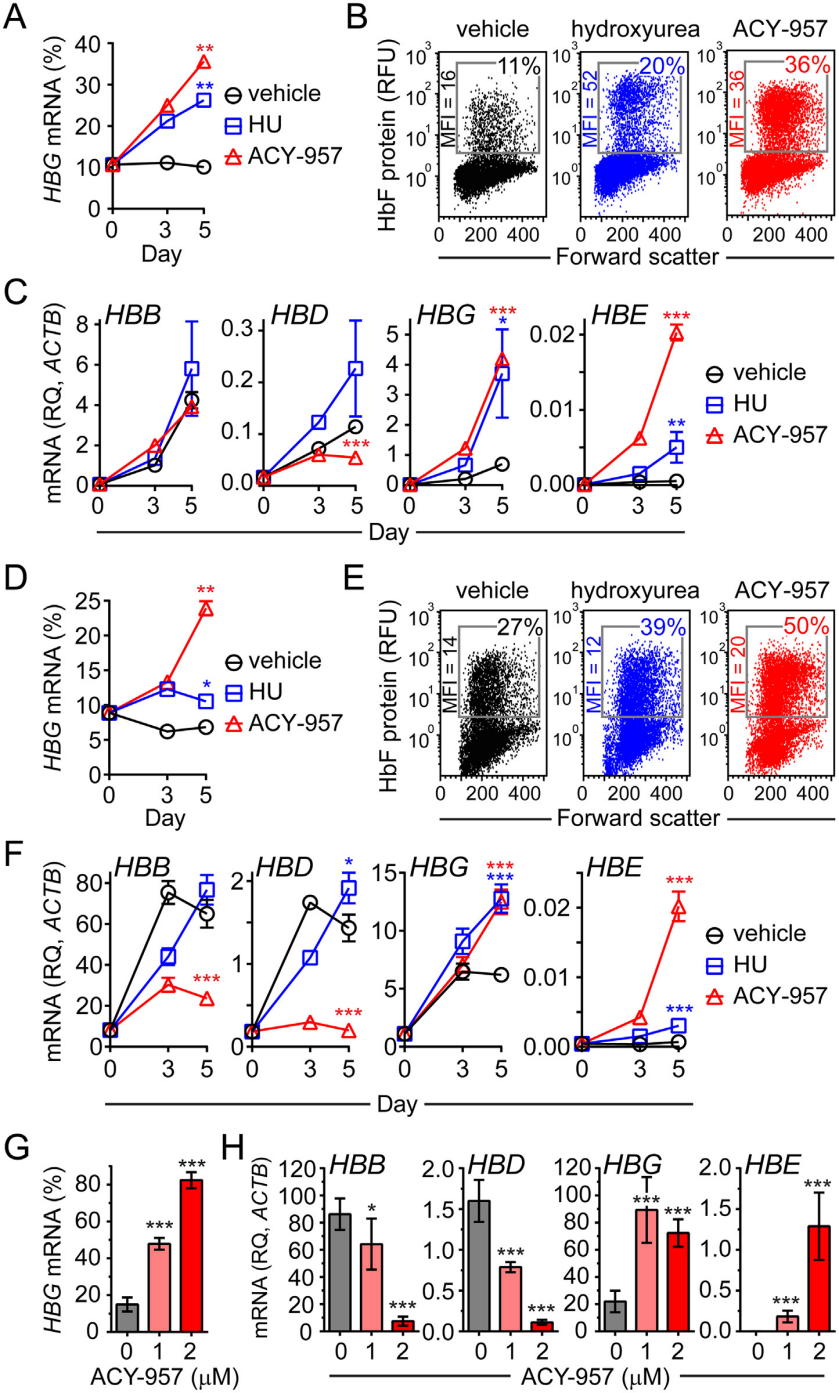
ACY-957 induces *HBG* mRNA and HbF protein in primary cells from healthy donors. (A) Time-dependent increase in the percent *HBG* mRNA in CS1 cells. BM cells were cultured in CS1 expansion media, then shifted to CS1 differentiation media for 5 days with vehicle (dimethyl sulfoxide), 30 μM hydroxyurea (HU), or 1 μM ACY-957 (mean ± SD, n = 2 QPCR and n = 2 cell culture replicates). Data is representative of experiments using cells from three independent donors. (B) HbF protein induction in ACY-957 treated cells. Cells from day 5 of differentiation in ‘A’ were stained with an anti-HbF antibody and detected by flow cytometry. (C) Effect of ACY-957 on each β-like globin transcript. Samples from ‘A’ with each β-like globin transcript plotted relative to β-actin (*ACTB*). (D) Time-dependent increase in the percent *HBG* mRNA in CS2 cells. BM cells were cultured in CS2 expansion media, then shifted to CS2 differentiation media for 5 days with vehicle, 30 μM HU, or 1 μM ACY-957 (mean ± SD, n = 2 QPCR and n = 2 cell culture replicates). Data is representative of experiments using cells from two independent donors. (E) HbF protein induction in ACY-957 treated cells. Cells from day 5 of differentiation in ‘D’ were stained with an anti-HbF antibody and detected by flow cytometry. (F) Samples from ‘D’ with each β-like globin transcript plotted relative to *ACTB*. (G) Dose-dependent increase in percent *HBG* mRNA in BFU-E colonies derived from human bone marrow mononuclear cells cultured with ACY-957 (mean ± SD, n = 3 cell culture replicates). Data is representative of experiments using cells from two independent donors. (H) Samples from ‘G’ with each β-like globin transcript plotted relative to *ACTB*. In panels ‘A’, ‘C’, ‘D’, and ‘F’, P-values were calculated on day 5 only using a two-tailed *t* test. In panel ‘G’ and ‘H’ P-values were calculated using a two-tailed *t* test. For all panels *P<0.05, **P<0.005, and ***P<0.0005 compared to vehicle treatment. *HBG* mRNA (%) = [*HBG*/(*HBB*+*HBD*+*HBG*+*HBE*)]*100. MFI, mean fluorescent intensity. RFU, relative fluorescence units. RQ, relative quantity.

ACY-957 at 1 μM was sufficient to induce histone acetylation (Fig 1E). Therefore, to determine if ACY-957 could induce *HBG* mRNA, differentiating CS1 or CS2 cells were cultured in the presence of 1 μM ACY-957 or 30 μM of hydroxyurea, a known *HBG* inducer. HDAC inhibition by ACY-957 led to a significant time-dependent induction in the percentage of *HBG* mRNA relative to total β-like globin mRNA in both culture systems which exceeded the response observed with hydroxyurea (Fig 2A and 2D). At day 5, ACY-957 treated cells had *HBG* levels of 35% and 24% in CS1 and CS2, respectively, representing an approximately 3.5-fold increase over vehicle treated controls.

To determine if the *HBG* mRNA induction observed in the presence of ACY-957 was associated with an increase in the HbF protein, CS1 and CS2 cells at day 5 were stained with a fluorescently conjugated anti-HbF antibody and evaluated by flow cytometry. HbF positive cells were increased by 2 to 3-fold upon treatment with ACY-957, which exceeded the response observed with hydroxyurea (Fig 2B and 2E). HDAC inhibition also increased the mean fluorescent intensity (MFI) of the HbF positive cells, a measure of HbF abundance per cell, by up to 2-fold. These findings suggest that *HBG* mRNA level is a reliable indicator of HbF protein level in these culture systems.

To understand how each individual β-like globin mRNA contributes to the observed increase in percent *HBG* resulting from ACY-957 treatment, each β-like globin mRNA was plotted relative to the housekeeping control β-actin (*ACTB*). ACY-957 treatment significantly increased levels of *HBE* and *HBG* mRNA and decreased levels of *HBD* and *HBB* mRNA (Fig 2C and 2F). Suppression of the adult globins, *HBB* and *HBD*, and induction of embryonic/fetal globins, *HBE* and *HBG*, is consistent with a model in which HDAC inhibition delays or reverses the process of globin switching.

To determine if ACY-957 could induce *HBG* in long-term culture, burst forming unit erythroid (BFU-E) colonies were grown in the presence of ACY-957 for 14 days. The percent *HBG* increased in a dose-dependent manner, from 15% in vehicle treated cells to 48% and 82% in cells treated with 1 and 2 μM ACY-957, respectively (Fig 2G). Consistent with the findings in CS1 and CS2, increases in percent *HBG* by ACY-957 were due to both increased *HBE* and *HBG* mRNA and decreased *HBD* and *HBB* mRNA, further supporting a globin switching model of activation (Fig 2H).

**Fig 3:**
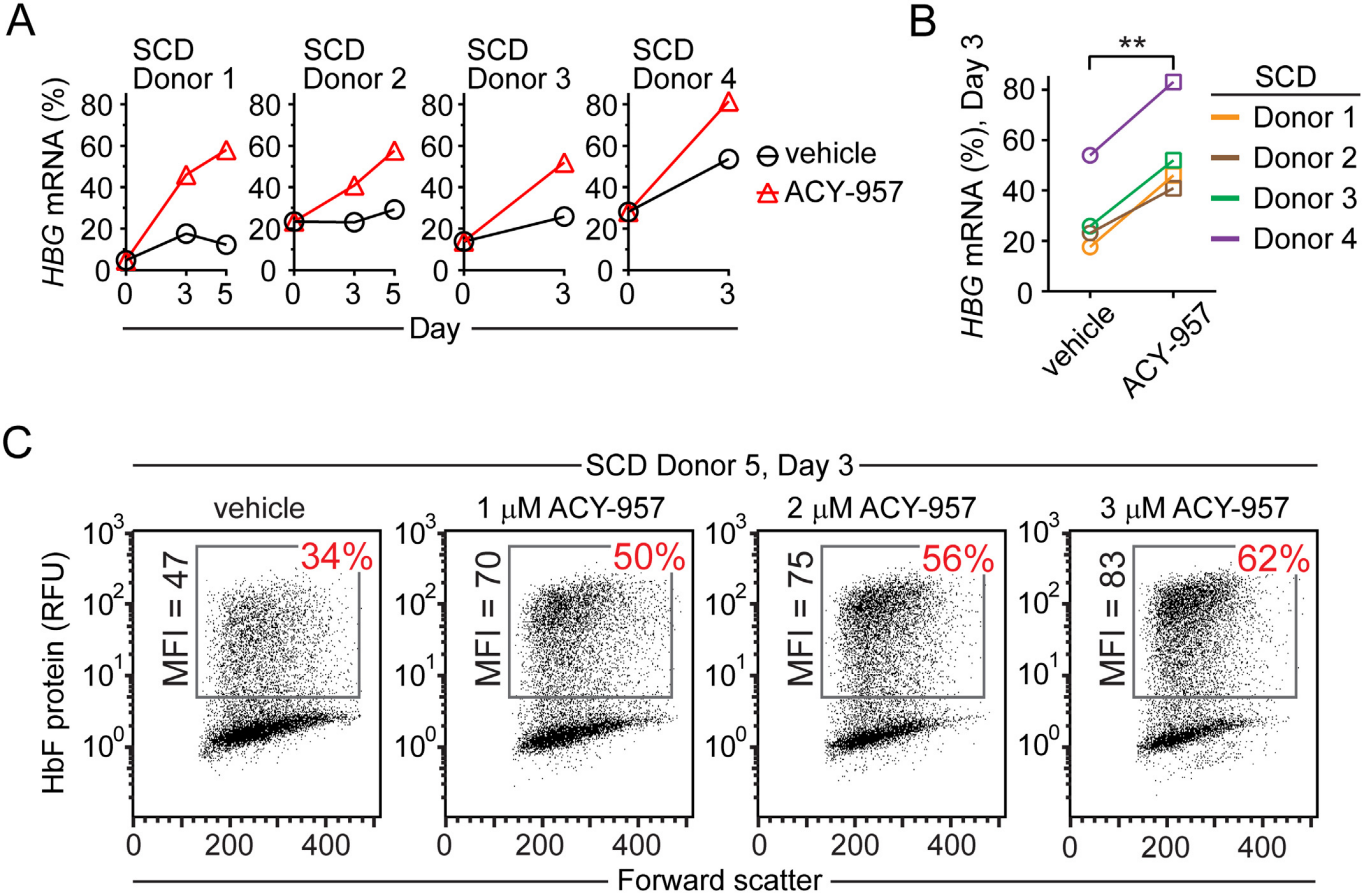
ACY-957 induces *HBG* mRNA and HbF protein in primary cells from sickle cell disease patients. (A) PBMC from four SCD donors were isolated, expanded, and then placed in differentiation media with vehicle or 1 μM ACY-957 for the indicated number of days. (B) Percent *HBG* in sickle cell patient cells in ‘A’ after 3 days of culture. (C) Hematopoietic progenitors were isolated from peripheral blood of a sickle cell patient, expanded, and then cultured for 3 days in differentiation media with 1, 2, or 3 μM ACY-957. HbF protein was detected by flow cytometry. P-values were calculated using a two-tailed, paired *t* test. **P<0.005 compared to vehicle treatment. *HBG* mRNA (%) = [*HBG*/(*HBB*+*HBD*+*HBG*+*HBE*)]*100.

To determine if ACY-957 could induce *HBG* in SCD patient samples, peripheral blood mononuclear cells (PBMC) from patients homozygous for the sickle cell mutation were cultured in CS1 expansion media, and then placed in differentiation media for 3 to 5 days in the presence of 1 μM of ACY-957 or vehicle. ACY-957 led to significantly elevated *HBG* in all four donor samples tested (Fig 3A and 3B). The largest induction was observed in donor 1, where *HBG* increased from 12% in vehicle control to 58% upon ACY-957 treatment. Furthermore, ACY-957 treatment led to a dose-dependent induction in the number of SCD donor cells staining positive for the HbF protein and in the HbF protein abundance per cell (Fig 3C).

### Effect of HDAC inhibition on erythroid maturation

**Fig 4:**
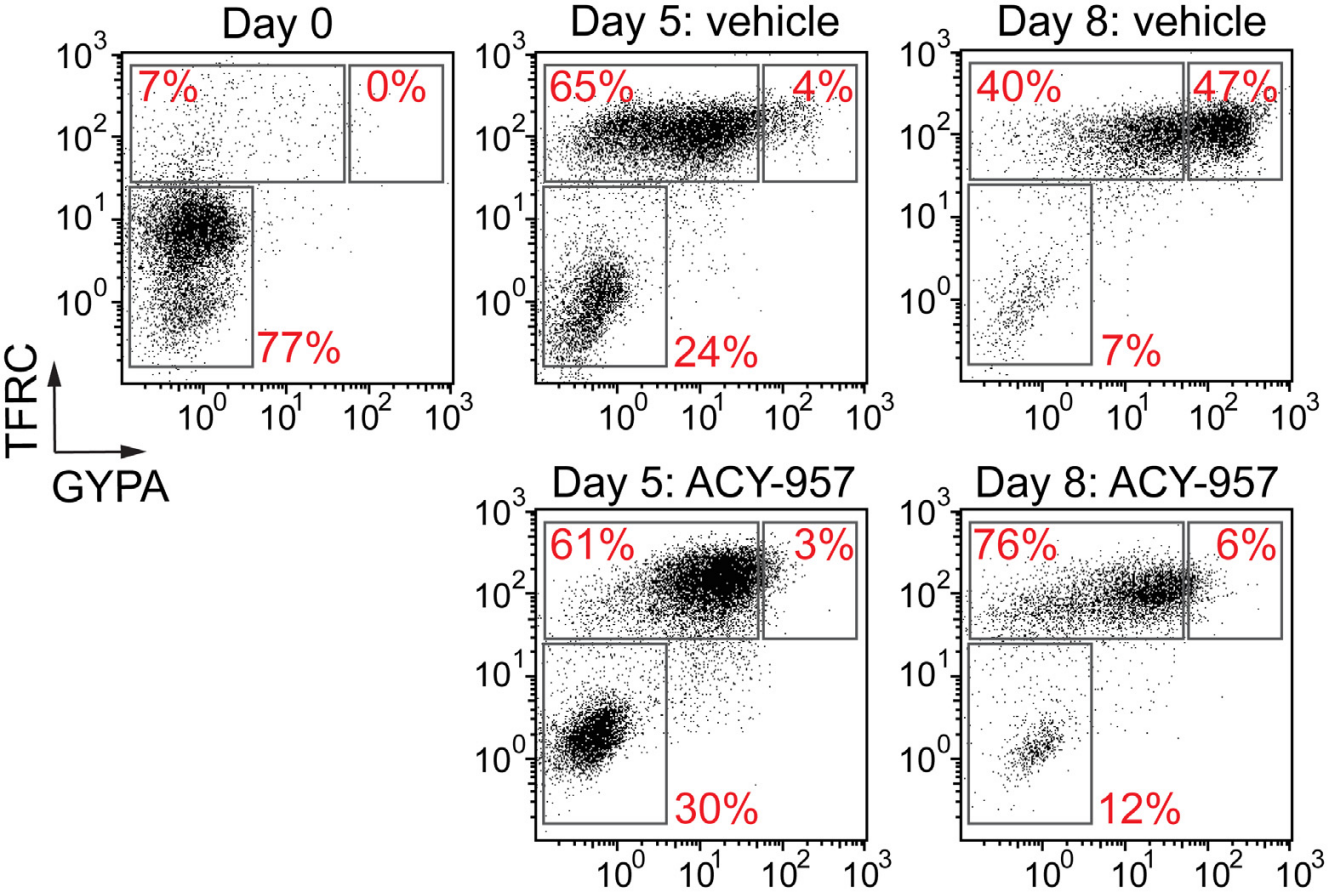
Effect of HDAC inhibition on erythroid differentiation. BM cells were cultured in CS1 expansion media, and then placed in CS1 differentiation media for 8 days with vehicle or 1 μM ACY-957. Erythroid maturation stage was determined by flow cytometry using the cell surface markers TFRC and GYPA. Representative of experiments performed with cells from four independent donors.

To investigate the effects of HDAC1/2 inhibition on erythroid maturation, we followed differentiation by flow cytometry using TFRC and GYPA [38]. BM cells were cultured in CS1 expansion media for 7 days, yielding a pool of TFRC^neg^GYPA^neg^ cells composed of primitive and more differentiated hematopoietic progenitors of multiple lineages (Fig 4, ‘day 0’) [39]. These cells were then shifted to CS1 differentiation media containing either 1 μM of ACY-957 or vehicle control. Cells treated with ACY-957 differentiated to the same extent as vehicle treated cells during the first 5 days, becoming TFRC^pos^GYPA^mid^ (Fig 4, ‘day 5’). The majority of vehicle control cells continued to differentiate over the next 3 days, becoming TFRC^pos^GYPA^pos^ by day 8. In contrast, cells treated with ACY-957 did not fully upregulate GYPA, but accumulated at the TFRC^pos^GYPA^mid^ stage (Fig 4, ‘day 8’). TFRC^pos^GYPA^mid^ cells are equivalent to proerythroblasts, while TFRC^pos^GYPA^pos^ cells include the more differentiated basophilic and polychromatic erythroblasts [38]. Therefore, HDAC1/2 inhibition using 1 μM of ACY-957 was able to inhibit differentiation of proerythroblasts to basophilic erythroblasts in the liquid culture systems utilized in this study.

### Effect of HDAC1/2 inhibition on gene expression

**Fig 5:**
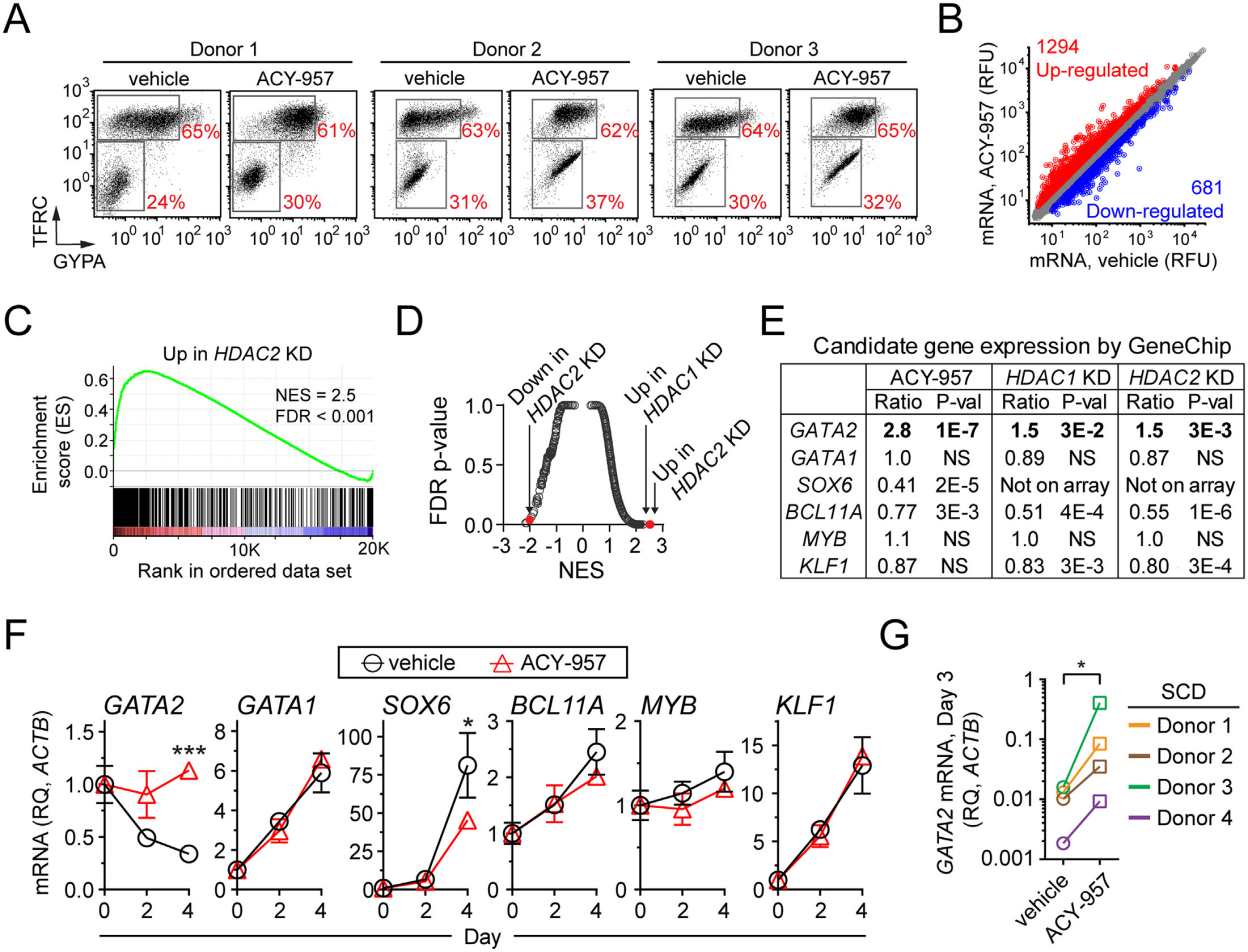
Effect of HDAC1/2 inhibition on gene expression in erythroid progenitors. (A) Erythroid maturation stage of cells used in gene expression profiling experiments. BM cells were cultured in CS1 expansion media and then shifted to CS1 differentiation media for 5 days with vehicle or 1 μM ACY-957. At the end of the culture period, erythroid maturation stage was determined by flow cytometry and total RNA was isolated for analysis using Affymetrix GeneChips. Experiments were performed using cells from three independent donors. (B) Differentially expressed genes resulting from ACY-957 treatment using a filter of absolute fold change greater than 1.5 and a P-value less than 0.025. (C) Gene set enrichment analysis demonstrates that genes up-regulated by HDAC2 knockdown (‘Up in *HDAC2* KD’ gene set) are significantly overrepresented at the top of a ranked list of gene expression changes resulting from ACY-957 treatment. Significant enrichment is illustrated by the positive running enrichment score (ES) marked by the green line, normalized enrichment score (NES) = 2.5, and false discovery rate (FDR) P-value < 0.001. (D) Enrichment scores of the ‘Up in *HDAC1* KD’, ‘Up in *HDAC2* KD’, and ‘Down in *HDAC2* KD’ gene sets relative to all gene sets (2777 total) in the Molecular Signatures Database collection of Chemical and Genetic Perturbations. (E) GeneChip derived gene expression ratios of candidate *HBG* modulators following ACY-957 treatment, *HDAC1* knockdown, or *HDAC2* knockdown. Ratios expressed as treatment versus control. NS, not significant. (F) Gene expression of candidate *HBG* modulators by QPCR. BM cells were cultured in CS1 expansion media, and then shifted to CS1 differentiation media for 4 days in presence of vehicle or 1 μM ACY-957. Gene expression is shown relative to *ACTB* and normalized to day 0 (mean ± SD, n = 3 cell culture replicates). P-values were calculated for day 4 using a two-tailed *t* test. *P<0.05 and ***P<0.0005 compared to vehicle treatment. Data is representative of experiments using cells from two independent donors. (G) Induction of *GATA2* mRNA by ACY-957 in cells from the four SCD donors described in Fig 3A. P-values were calculated using a two-tailed, paired *t* test. *P<0.05 compared to vehicle treatment.

We interrogated the mechanism through which HDAC1/2 inhibition induces *HBG* by performing gene expression profiling on ACY-957 or vehicle treated cells in CS1. Because HDAC inhibition prevented cells from fully upregulating GYPA (Fig 4) we isolated RNA at day 5 of differentiation, a time point prior to the observed differentiation block. For each of 3 independent experiments, vehicle and ACY-957 treated cells showed similar TFRC/GYPA differentiation profiles (Fig 5A), lending confidence that the resulting gene expression profiles were measuring a compound-specific effect that was not confounded by a shift in the maturity of cell populations. We found ACY-957 treatment induced twice as many genes as it suppressed, 1294 and 681 respectively, a result consistent with the positive association of histone acetylation with chromatin accessibility and gene expression (Fig 5B).

To determine if the gene expression changes resulting from ACY-957 treatment were similar to the gene expression changes resulting from *HDAC1* or *HDAC2* knockdown (KD), we analyzed published gene expression data for *HDAC1* or *HDAC2* KD in primary erythroblasts [21], and appended these gene sets to a list of pre-existing gene sets. This collection of 2781 gene sets was queried against the ACY-957 and vehicle expression profiles. Robust and statistically significant enrichment was identified for the gene set ‘Up in *HDAC2* KD’ (Fig 5C), as well as for the gene sets ‘Up in *HDAC1* KD’ and ‘Down in *HDAC2* KD’. In other words, genes up-regulated by *HDAC1* or *HDAC2* KD are significantly overrepresented at the top of a ranked list of fold changes resulting from ACY-957 treatment. Finally, as a measure of biological specificity, false discovery rate was plotted as a function of normalized enrichment score for all 2781 gene sets (Fig 5D). ‘Up in *HDAC1* KD’ and ‘Up in *HDAC2* KD’ were the top two enriched gene sets in the ACY-957 expression profile. Taken together, these finding suggest that pharmacologic inhibition of HDAC1/2 recapitulates genetic ablation of *HDAC1* or *HDAC2*.

Next, using the GeneChip data, we took a candidate gene approach to determine which *HBG* modulators were changing as a result of both chemical and genetic HDAC1/2 inhibition (Fig 5E). We found that the *HBG* repressors *BCL11A* [24] and *SOX6* [40], were down-regulated 1.3- and 2.5-fold by ACY-957 treatment, respectively. *BCL11A* was also suppressed 2-fold by *HDAC1* or *HDAC2* KD. In contrast, other *HBG* repressors, such as *MYB* [41, 42] and *KLF1* [43, 44] were unaffected. Expression changes were not observed for other genes involved in *HBG* regulation, including *KDM1A* [13], *NR2C1*, *NR2C2* [45], *NR2F2* and nuclear factor Y subunits [46], and proteins associating with *BCL11A* [22] (data not shown). However, *GATA2*, a proposed *HBG* and *HBE* activator [47, 48], was up-regulated 2.8-fold by ACY-957 treatment and 1.5-fold by knockdown of *HDAC1* or *HDAC2*.

These observations were confirmed and extended using QPCR to measure temporal gene expression changes resulting from 1 μM ACY-957 treatment in CS1 (Fig 5F). In control cells, *GATA1* increased and *GATA2* decreased during the 4 day differentiation period, a result consistent with the known expression pattern of these genes during erythropoiesis. However, ACY-957 treatment prevented the suppression of *GATA2*, resulting in a 3.3-fold increase relative to control cells at day 4. In contrast, *GATA1* and *KLF1*, master regulators of erythropoiesis, were unaffected by ACY-957 treatment (Fig 5F), a finding consistent with the observed similarity in TFRC/GYPA profiles between ACY-957 and vehicle treated cells at day 5 (Fig 4), and further suggesting differentiation is unaffected by ACY-957 treatment during the first 5 days of culture in CS1. Furthermore, unlike *BCL11A* and *SOX6* suppression, *GATA2* induction by ACY-957 was observed as early as day 2 and correlated with *HBG* induction. Finally, we observed elevated *GATA2* expression in primary cells derived from SCD patients following 3 days of culture with 1 μM ACY-957 (Fig 5G). Taken together, these results suggest that inhibition of HDAC1/2 prevents the suppression of *GATA2* gene expression during the early stages of erythroid maturation, and raised the possibility that GATA2 may act as an *HBG* activator.

### GATA2 overexpression induces *HBG* and suppresses *HBB*

**Fig 6:**
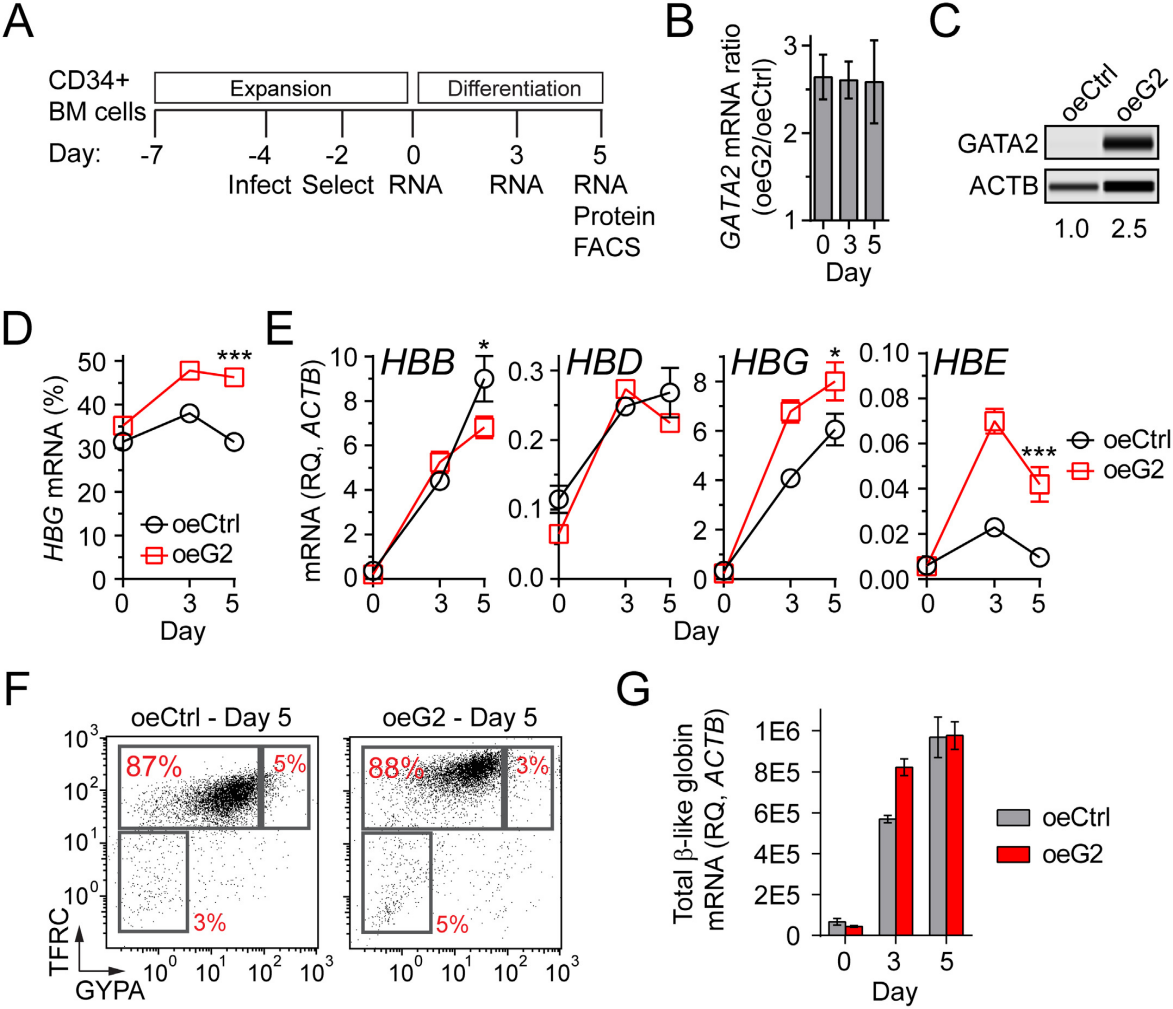
Overexpression of GATA2 induces *HBG* and suppresses *HBB* in erythroid progenitors. (A) Experimental design. BM cells were cultured for 3 days in CS1 expansion media, and then infected with lentivirus containing GFP (oeCtrl) or full length *GATA2* transcript (oeG2). Infected cells were selected for by addition of puromycin to the culture for 2 days. Cells were then shifted to CS1 differentiation media for the remainder of the experiment. (B) *GATA2* mRNA levels in oeG2 cells expressed relative to oeCtrl (mean ± SD, n = 2 QPCR replicates for each of n = 2 infection replicates). (C) GATA2 protein levels at day 5 of differentiation. Values represent GATA2 protein levels relative to ACTB and then normalized to oeCtrl. (D) *HBG* mRNA levels during differentiation of oeCtrl and oeG2 cells (mean ± SD, n = 2 QPCR replicates for each of n = 2 infection replicates). *HBG* mRNA (%) = [*HBG*/(*HBB*+*HBD*+*HBG*+*HBE*)]*100. (E) Samples from ‘D’ with each β-like globin transcript expressed relative to *ACTB* mRNA. (F) TFRC and GYPA cell surface expression at day 5 of differentiation. (G) Total β-like globin mRNA (sum of *HBB*, *HBD*, *HBG*, and *HBE*) in samples from ‘D’. For panels ‘D’, ‘E’, and ‘G’, P-values were calculated on day 5 using a two-tailed *t* test. *P<0.05, **P<0.005, ***P<0.0005 for oeG2 compared to oeCtrl. Data is representative of experiments performed using cells from three independent donors.

To test the hypothesis that GATA2 is an *HBG* activator, full length *GATA2* or green fluorescent protein were lentivirally delivered to CS1 expanded cells and then placed in CS1 differentiation media (Fig 6A). In cells with ectopic GATA2 (oeG2), *GATA2* mRNA was 2.5-fold higher than green fluorescent protein control cells (oeCtrl) throughout the differentiation period (Fig 6B). Consistent with this finding, GATA2 protein level in oeG2 cells was 2.5-fold greater than in oeCtrl cells at day 5 (Fig 6C). Overexpression of GATA2 significantly increased the percent *HBG* relative to control cells at day 3 and 5 of differentiation (Fig 6D). Interrogation of each individual β-like globin transcript relative to *ACTB* revealed that the elevated percent *HBG* in oeG2 cells resulted from increased *HBG* mRNA and decreased *HBB* mRNA (Fig 6E). GATA2 overexpression also significantly increased *HBE* mRNA. These findings are consistent with observations in cells treated with ACY-957 (Fig 2C, 2F and 2H) and suggest a globin switching model of *HBG* activation in response to elevated GATA2.

Altering normal levels of GATA2 has the potential to affect erythroid differentiation, which could confound the interpretation of the finding above. Therefore, we measured cell surface levels of TFRC and GYPA by flow cytometry in oeCtrl and oeG2 cells at day 5 (Fig 6F). We found their TFRC/GYPA profiles to be highly similar, with the majority of cells upregulating both markers to a similar extent with each treatment. As an additional indicator of erythroid differentiation stage, we measured the total β-like globin mRNA levels in control and GATA2 overexpressing cells. Consistent with the TFRC/GYPA profiles, we found little difference in the total level of β-like globin mRNA at day 5 (Fig 6G). Taken together, our data suggests that elevated GATA2 expression in erythroid progenitors is sufficient to induce *HBG*, without overtly affecting their maturation during the 5 day differentiation period evaluated here.

### GATA2 knockdown attenuates *HBG* induction by ACY-957

**Fig 7:**
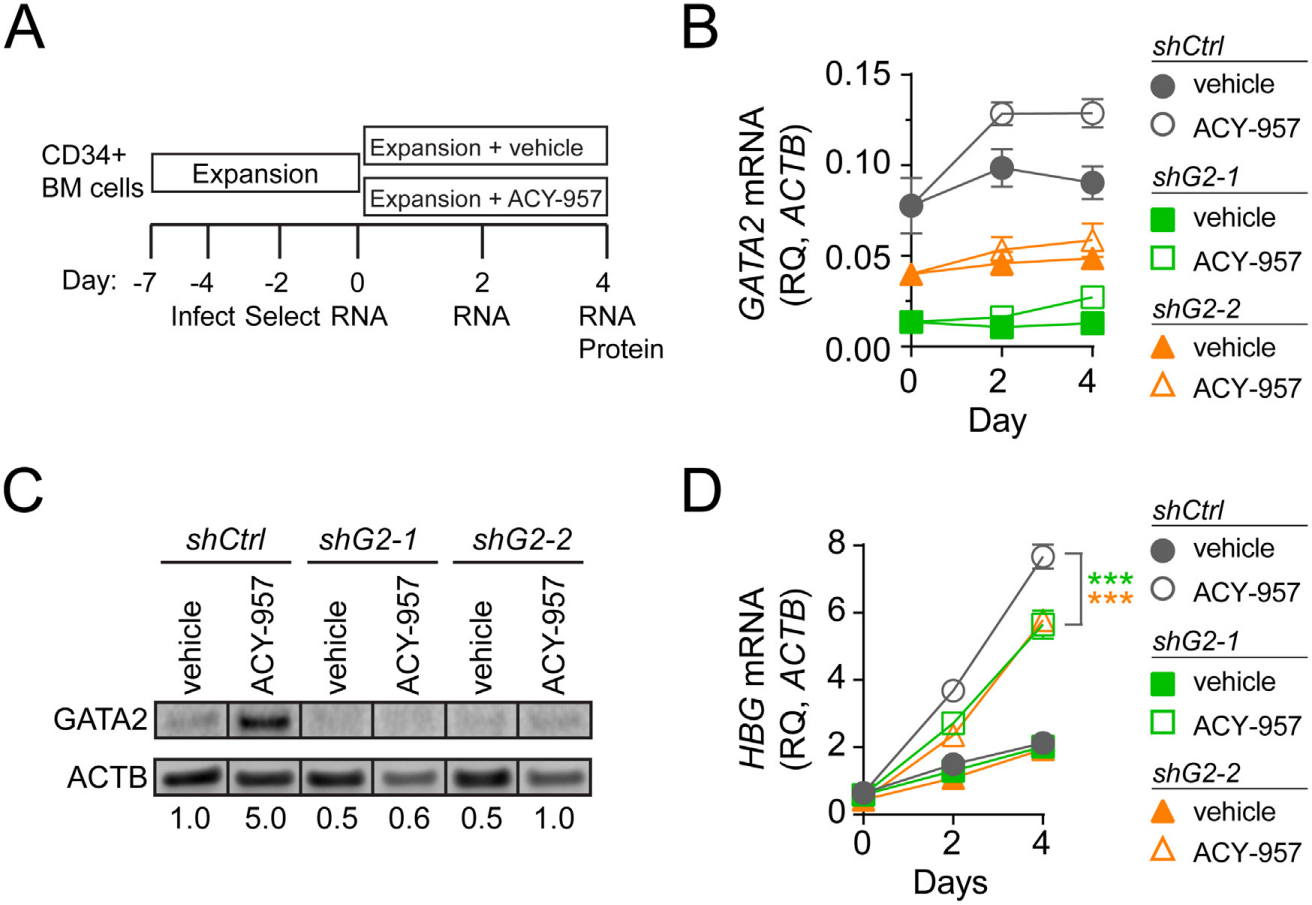
*GATA2* knockdown attenuates *HBG* induction by ACY-957. (A) Experimental design. BM cells were cultured for 3 days in CS1 expansion media, and then infected with lentivirus containing a control hairpin that does not target any human gene (shCtrl) or with two different *GATA2* targeting hairpins (shG2-1, shG2-2). Infected cells were selected by addition of puromycin to the culture for 2 days. Following removal of puromycin at day 0, cells expressing each hairpin were cultured for 4 days in CS1 expansion media with 1 μM ACY-957 or vehicle control. (B) *GATA2* mRNA levels were measured by QPCR and expressed relative to *ACTB* mRNA (mean ± SD, n = 2 QPCR replicates for each of n = 2 infection replicates). (C) GATA2 protein levels at day 4. Values represent GATA2 protein levels normalized to ACTB and relative to vehicle treated shCtrl. (D) *HBG* mRNA levels were measured by QPCR and expressed relative to *ACTB* mRNA (mean ± SD, n = 2 QPCR replicates for each of n = 2 infection replicates). For panel ‘D’ P-values were calculated on day 4 using a two-tailed *t* test, ***P<0.0005.

Next we sought to determine if GATA2 is necessary for *HBG* induction by ACY-957. Short hairpin RNA targeting *GATA2* (shG2-1 or shG2-2), or a non-targeting control (shCtrl) were lentivirally delivered to CS1 expanded cells (Fig 7A). Following puromycin selection, shRNA-containing cells were cultured for an additional 4 days in CS1 expansion media containing either 1 μM ACY-957 or vehicle control. Since *GATA2* levels decline during erythroid differentiation (Fig 5F), knockdown experiments were performed entirely in CS1 expansion media, which supports hematopoietic progenitors and maintained *GATA2* mRNA at a constant level in shCtrl cells (Fig 7B, solid gray circles).

In vehicle treated cells, *GATA2* mRNA was reduced by 85% or 50% by shG2-1 or shG2-2 hairpins, respectively, relative to shCtrl hairpin throughout the 4 day culture period (Fig 7B). Consistent with this finding, GATA2 protein was reduced by 50% by shG2-1 and shG2-2 hairpins relative to shCtrl hairpin in vehicle treated cells at day 4 (Fig 7C). As expected, treatment of shCtrl cells with ACY-957 led to increased GATA2 mRNA and protein (Fig 7B and 7C), that was coincident with a time-dependent increase in *HBG* mRNA (Fig 7D, compare gray solid circles to open circles). Treatment of shG2-1 and shG2-2 cells with ACY-957 also led to an increase in *HBG* mRNA (Fig 7D). However, the magnitude of this induction was reduced by 25% relative to the induction observed in shCtrl cells. This result suggests that reduced levels of GATA2 can attenuate *HBG* induction by ACY-957, further supporting a role for GATA2 in *HBG* activation.

### HDAC1 and HDAC2 co-occupy the *GATA2* locus

**Fig 8:**
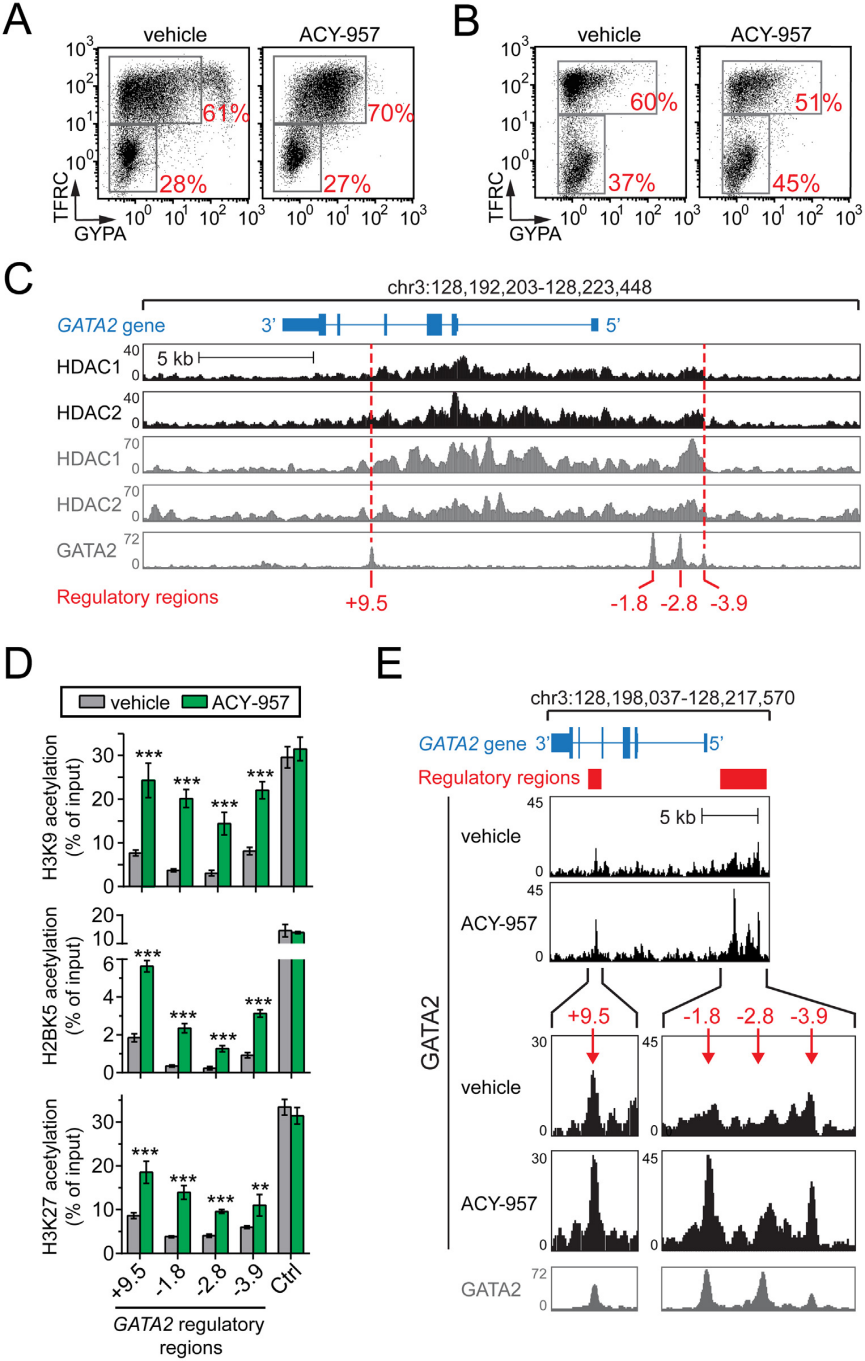
HDAC1/2 inhibition by ACY-957 leads to elevated levels of histone acetylation and GATA2 binding at *GATA2* regulatory regions. BM cells were cultured in CS1 expansion media and then shifted to CS1 differentiation media with vehicle or 1 μM ACY-957 until reaching a differentiation stage similar to cells used in the GeneChip experiments of Fig 5. (A) Erythroid differentiation stage of cells used for ChIP in Fig 8C, 8E and Fig 9A. (B) Erythroid differentiation stage of cells used for ChIP in Fig 8D and Fig 9B. (C) HDAC1 and HDAC2 ChIP-Seq profiles at the *GATA2* locus in erythroid progenitors. Chromatin was immunoprecipitated and sequenced using antibodies against HDAC1 or HDAC2 (black histogram tracks). Sequencing read count (y-axis) is plotted as a function of genomic region bin (x-axis). Publically available ENCODE Consortium ChIP-Seq data for HDAC1, HDAC2, and GATA2 in K562 cells is shown (gray histogram tracks) [49, 50]. The previously described *GATA2* enhancer regions (red text) map to GATA2 binding peaks in K562 cells. In both K562 and primary erythroid progenitors, HDAC1 and HDAC2 occupy a region bounded by the +9.5 kb and -3.9 kb enhancer regions (red dashed lines). (D) Histone acetylation at *GATA2* regulatory regions in vehicle and ACY-957 treated erythroid progenitors. Chromatin from each treatment was immunoprecipitated using anti-H3K9ac, anti-H2BK5ac, or anti-H3K27ac antibodies. A region near the INO80 gene (Ctrl), identified as a region of saturated acetylation across a wide variety of ENCODE cell lines, was used as a control (mean ± SD, n = 2 QPCR replicates for each of n = 3 IP replicates per antibody). P-values were calculated using a two-tailed *t* test. **P<0.005 and ***P<0.0005 for ACY-957 compared to vehicle control. (E) GATA2 binding at *GATA2* regulatory regions following ACY-957 treatment. IP with anti-GATA2 antibody and sequencing as described in ‘C’. ACY-957 treatment resulted in increased GATA2 occupancy (black histogram tracks) at the *GATA2* regulatory regions, indicated by solid red bars (wide view) or red arrows (magnified view). ACY-957-responsive regions localize to GATA2 binding peaks in K562 cells (gray histogram track).

To investigate how HDAC1/2 inhibition drives GATA2 activation, we performed a series of chromatin immunoprecipitation (ChIP) experiments. BM cells were expanded in CS1, and then shifted to CS1 differentiation media in the presence of vehicle or 1 μM ACY-957 for several days, yielding erythroid progenitor cells with TFRC/GYPA differentiation profiles similar to those used for GeneChip experiments and unaffected by compound treatment (Fig 8A and 8B).

ChIP followed by next generation sequencing (ChIP-Seq) was performed on vehicle treated cells using antibodies against HDAC1 or HDAC2. We found that HDAC1 and HDAC2 are both highly abundant within a 15 kilobase (kb) region of the *GATA2* locus (Fig 8C, black histograms), beginning in a region 5 kb upstream of the hematopoietic-specific transcription start site and ending between the 4^th^ and 5^th^ exon, 10 kb downstream of the transcription start site. The strong correlation of HDAC1 and HDAC2 binding peaks suggests that they co-occupy this region.

We also compared our data to publically available ENCODE ChIP-seq data from the erythroleukemia cell line K562 (Fig 8C, gray histograms), which is known to express a high level of *HBG* mRNA and a low level of *HBB* mRNA [49, 50]. We observed that HDAC1/2 occupancy at the *GATA2* locus is tightly correlated between K562 and our primary erythroid progenitors. Furthermore, we observed that this region of HDAC1/2 occupancy is bounded by the previously described +9.5 and -3.9 kb regulatory regions of the murine *Gata2* gene [51, 52]. These regulatory regions are clearly identified by GATA2 binding peaks in K562 cells (Fig 8C, red lines) and were confirmed by alignment of the murine +9.5 and -3.9 kb regulatory sequences to the human genome. This region of HDAC1/2 occupancy also includes the -2.8 kb and -1.8 kb *GATA2* regulatory regions [52].

### ACY-957 increases histone acetylation and GATA2 binding at *GATA2* regulatory regions

GATA2 is known to be activated by a positive auto-regulatory loop in which GATA2 binding at the +9.5, -1.8, -2.8, and -3.9 kb regulatory regions plays a key role [51–54]. The replacement of GATA2 by GATA1 at these regulatory regions, a process referred to as ‘gata switching’, results in the suppression of *GATA2* gene expression during early stages of erythroid cell maturation. GATA2 silencing is associated with a decrease in histone acetylation [51] and chromatin accessibility [55] at the +9.5, -1.8, -2.8, and -3.9 kb sites. Therefore, we hypothesized that ACY-957-mediated inhibition of HDAC1/2 was leading to increased histone acetylation at these important regulatory regions, thereby promoting or prolonging *GATA2* gene expression.

To test this hypothesis, ChIP followed by QPCR (ChIP-QPCR) was performed on vehicle or ACY-957 cells using antibodies against acetylated H2BK5, H3K9, and histone H3 lysine 27 (H3K27). In chromatin state maps these histone modifications have been associated with active regulatory regions, such as enhancers and promoters of actively transcribed genes [50, 56–58]. We found that ACY-957 treatment led to significant increases in histone acetylation at the +9.5, -1.8, -2.8, and -3.9 kb *GATA2* regulatory regions, with maximum increases of 4- to 8-fold at the -1.8 kb region (Fig 8D).

To see if the increases in histone acetylation were associated with increases in GATA2 binding, we performed GATA2 ChIP-Seq in vehicle or ACY-957 treated cells. We observed that GATA2 occupancy at the *GATA2* locus is tightly correlated between K562 and our primary erythroid progenitors (Fig 8E, compare black and gray histograms). In both cases, GATA2 binding was limited to the +9.5, -1.8, -2.8, and -3.9 kb regulatory regions. In response to ACY-957 treatment, GATA2 protein showed increased binding at all *GATA2* regulatory regions, with a maximum increase in peak height of 3-fold at the -1.8 kb region. These experiments demonstrate that ACY-957 treatment results in elevated histone acetylation and GATA2 occupancy at *GATA2* enhancer sites, suggesting that HDAC1/2 inhibition by ACY-957 maintains the activity of the GATA2 autoregulatory loop, which is normally inactivated during erythroid maturation.

### ACY-957 increases GATA2 binding at a region near the *HBD* promoter

**Fig 9:**
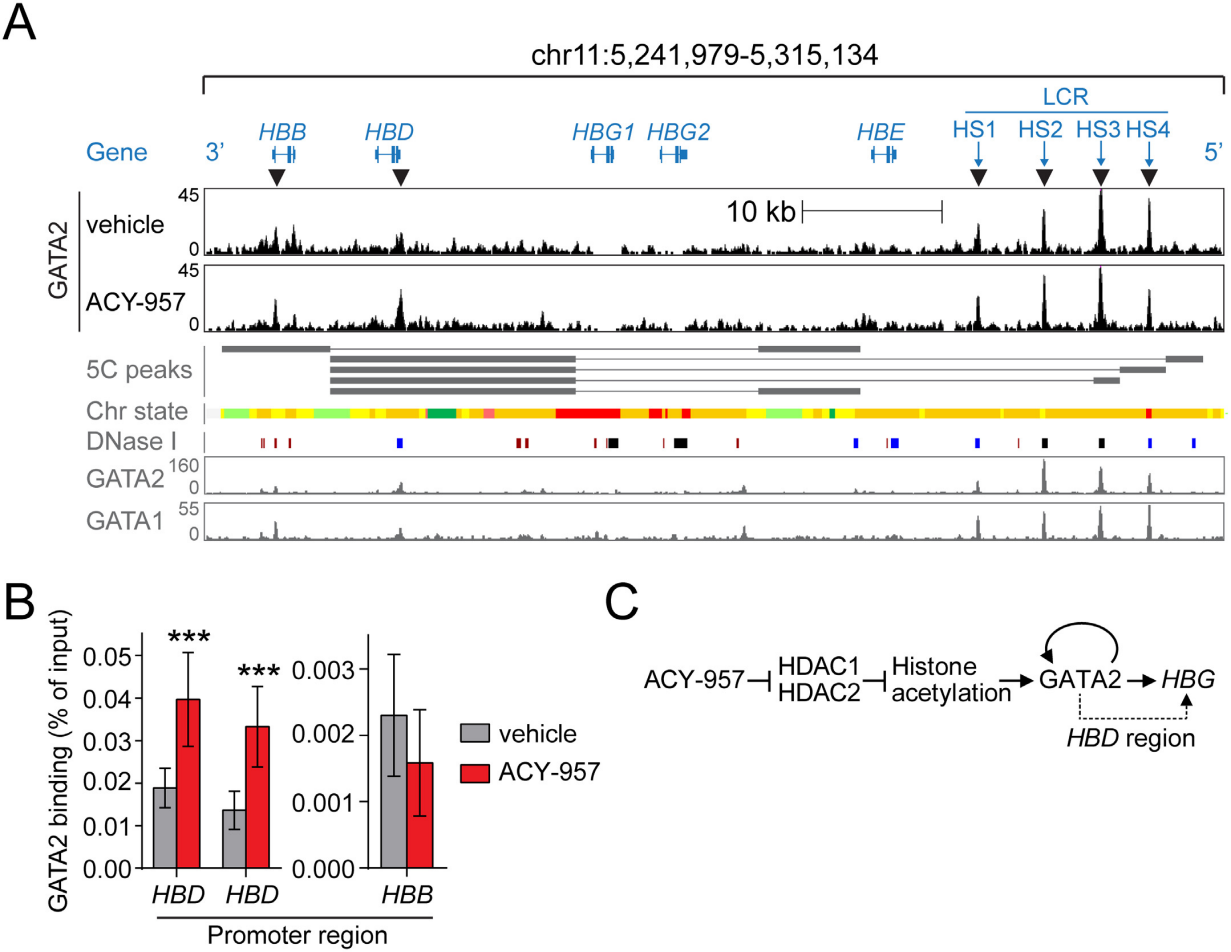
HDAC1/2 inhibition by ACY-957 leads to elevated levels of GATA2 binding at the *HBD* promoter. Changes in GATA2 binding due to ACY-957 treatment were surveyed across the 70 kb β-like globin gene cluster. Cell culture, IP with anti-GATA2, and sequencing were performed as described in Fig 8. (A) The location of each β-like globin gene and LCR hypersensitivity sites (HS1-4) are shown in the ‘Gene’ track (blue). GATA2 occupancy is shown for vehicle or ACY-957 treated primary erythroblasts (‘vehicle’ and ‘ACY-957’ tracks, black). Only 6 regions had statistically significant GATA2 binding peaks in either vehicle or ACY-957 treated cells (indicated by triangles below the ‘Gene’ track). Within the 70 kb β-like globin locus, ACY-957 treatment lead to elevated levels of GATA2 binding only at a single region, found at the *HBD* promoter. ENCODE Consortium data for K562 cells shows this *HBD* region makes long-range looping interactions with the LCR (‘5C peaks’ track, gray), is a predicted enhancer (‘Chr state’ track, gray), is a region of open chromatin (‘DNase I’ track, gray), and is a region of GATA2 and GATA1 binding (‘GATA2’ and ‘GATA1’ tracks, gray). (B) Confirmation of GATA2 ChIP-Seq results by ChIP-QPCR. Two unique primer sets were used to detect GATA2 occupancy at the *HBD* promoter. A primer set at the *HBB* promoter, predicted by ChIP-Seq as a region where GATA2 had statistically significant binding that was unaltered by ACY-957 treatment, was used as a control (mean ± SD, n = 3 QPCR replicates for each of n = 3 IP replicates). P-values were calculated using a two-tailed *t* test. ***P<0.0005 for ACY-957 compared to vehicle control. (C) Summary of findings. ACY-957 inhibits HDAC1/2 leading to elevated histone acetylation at *GATA2* enhancer regions. Increased histone acetylation promotes occupancy of GATA2 at these regulatory regions, resulting in sustained activation of GATA2 during erythroid maturation through a positive autoregulatory loop. Elevated GATA2 contributes to *HBG* induction through an unknown mechanism, but may involve increased GATA2 binding at the *HBD* promoter (dashed line).

The results presented here suggest that GATA2 is a fetal globin activator, but the precise mechanism by which GATA2 regulates *HBG* remains unknown. GATA2 could affect *HBG* indirectly, through altering the expression of other factors which, in turn, act directly at the β-like globin gene cluster. While this possibility cannot be ruled out, the tight temporal correlation between *GATA2* and *HBG* induction in response to ACY-957 treatment suggests that GATA2 may act directly at the β-globin locus. To investigate this possibility, we looked at the β-like globin gene cluster in the GATA2 ChIP-Seq data presented above. Six statistically significant GATA2 binding peaks were identified in vehicle treated cells across the 70 kb region (Fig 9A); corresponding to four locus control region (LCR) hypersensitivity sites, a region in the *HBB* gene, and a region near the *HBD* gene promoter. These peaks aligned perfectly with GATA2 and GATA1 binding peaks in K562 cells from the ENCODE consortium. Upon treatment with ACY-957, GATA2 binding increased 1.8-fold at the *HBD* promoter region, while the other 5 regions were not affected (Fig 9A). An independent ChIP-QPCR experiment confirmed that ACY-957 treatment increases GATA2 binding by 2-fold at the *HBD* promoter, while a control region within the *HBB* gene was unaffected (Fig 9B).

## Discussion

Non-selective histone deacetylase inhibitors, such as MS-275, vorinostat, givinostat, and romidepsin, are known to induce HbF in cell lines and primary cells [16, 18, 23, 59, 60]. Several studies have now shown that genetic knockdown of *HDAC1* or *HDAC2* is sufficient to induce HbF [22, 23, 26]. Therefore, we designed ACY-957, an HDAC1/2-selective inhibitor, with the goal of inducing HbF while minimizing potential toxicity associated with these less selective HDAC inhibitors. We showed that ACY-957 is approximately 100-fold more potent against HDAC1/2 compared to HDAC3, and does not inhibit other HDAC isoforms (Fig 1). ACY-957 treatment induced *HBG* mRNA and HbF protein in a variety of primary erythroid progenitor cell culture systems, including cells derived from sickle cell patients (Figs 2 and 3). In side-by-side comparisons, *HBG* induction by ACY-957 compared favorably with hydroxyurea, the only approved therapy for sickle cell anemia (Fig 2).

Previous studies have shown that the HDAC inhibitors romidepsin and trichostatinA lead to an accumulation CD36^high^GYPA^mid^ erythroid progenitors by suppressing the EPO-mediated survival of CD36^high^GYPA^high^ cells [60]. In agreement, we found ACY-957 treatment led to an accumulation of TFRC^high^GYPA^mid^ cells (Fig 4), suggesting that HDAC1/2 inhibition alone is able to block the development of proerythroblasts to basophilic erythroblasts. Genetic models also support this finding, since monoallelic expression of *HDAC2* in an *HDAC1* knockout mouse led to a significant reduction in basophilic and polychromatic erythroblasts [61]. However, monoallelic expression of *HDAC1* in an *HDAC2* knockout animal had no effect on erythropoiesis, leading the authors to conclude that HDAC1 plays a more dominant role during erythroid development. Taken together, these findings suggest that future drug development should focus on identifying compounds with HDAC2-selectively, in order to minimize HDAC1-mediated erythroblast toxicity. Furthermore, our results suggest that intermittent dosing of ACY-957 will likely be required in future *in vivo* studies to allow maturation of erythroid progenitors and erythroblasts containing elevated HbF.

GeneChip experiments demonstrated that chemical inhibition or genetic knockdown of HDAC1/2 upregulates *GATA2* (Fig 5), an essential hematopoietic transcription factor expressed in a variety of cell types, including erythroid progenitors [62–65]. Previous work suggested that GATA2 may be involved in HbF activation, since *HBG* and *HBE* mRNA is increased in K562 cell lines overexpressing GATA2 [47], and because *GATA2* mRNA is more abundant in primary erythroid cells cultured under conditions that also yield elevated levels of HbF [66]. Our work demonstrated that overexpression of GATA2 in primary erythroid progenitors induces *HBG* and *HBE* (Fig 6) and knockdown of GATA2 attenuated *HBG* induction by ACY-957 (Fig 7). Both of these effects were modest, suggesting that HDAC1/2 inhibition affects *HBG* levels through mechanisms in addition to GATA2. Nonetheless, our results demonstrate that GATA2 is a *bona fide HBG* activator, and elevated levels of GATA2 are at least partially responsible for *HBG* induction by ACY-957.

Concurrent down-regulation of GATA2 and up-regulation of GATA1 is a hallmark of erythroid maturation in BFU-E, CFU-E, and proerythroblasts. As GATA1 levels rise, it replaces GATA2 at enhancer regions which control GATA2 expression. This exchange, the archetype of a prevalent gene regulatory mechanism known as gata-switching, is associated with the presence of the HDAC1/2-containing nucleosome remodeling and deacetylase (NuRD) complex, decreased histone acetylation, decreased GATA2 binding, and *GATA2* gene silencing [51, 52, 67, 68]. HDAC1/2 inhibition by ACY-957 appears to prevent this process, as we observed persistence of GATA2 expression that was associated with increased histone acetylation and increased GATA2 binding at enhancer regions involved in the positive autoregulation of the *GATA2* gene (Fig 8). In agreement with our data, GATA2 is highly upregulated and GATA1 remains unchanged when recruitment of the NuRD complex to sites of GATA1 binding is abolished in primary erythroblasts [68, 69]. Together, these data suggest that HDAC1/2 activity is required for GATA2 silencing during erythropoiesis, possibly acting to facilitate gata-switching at enhancer regions involved in *GATA2* gene regulation.

Our finding that ACY-957 increases GATA2 expression has therapeutic implications beyond hemoglobinopathies, as *GATA2* haploinsufficiency has been linked to a variety of disorders, including myelodysplastic syndrome (MDS) and acute myeloid leukemia (AML) [70, 71]. A causal link between epigenetic silencing of GATA2 and AML was recently shown, where re-expression of GATA2 resulted in the differentiation of leukemic stem cells and prevention of lethal AML *in vivo* [72]. Therefore, induction of GATA2 by ACY-957 represents a compelling molecular rationale for investigating the utility of an HDAC1/2-selective inhibitor in MDS and AML.

Our data support a model (Fig 9C) in which ACY-957 inhibits HDAC1/2, leading to elevated histone acetylation and elevated GATA2 binding at *GATA2* enhancer regions, resulting in sustained GATA2 expression during erythroid maturation. Elevated GATA2 resulting from HDAC1/2 inhibition contributes to the observed *HBG* induction through an unknown mechanism, but may involve increased GATA2 binding at the *HBD* promoter (Fig 9). A role for the *HBD* promoter region in regulating *HBG* expression is supported by genetic studies comparing β0 thalassemia, δβ thalassemia, and hereditary persistence of fetal hemoglobin patient samples [73, 74]. Furthermore, ENCODE data for K562 cells, which primarily express the *HBG* and *HBE* β-like globins, also implicate this region in regulating *HBG* expression, since it is marked as an active enhancer, open chromatin region, GATA2/GATA1 binding region, and makes significant looping interactions with the LCR (Fig 9A). Since the *HBD* promoter region is co-occupied by GATA1, SOX6, BCL11A, and the chromatin looping factor LDB1 [22, 40, 75], and because GATA2 and GATA1 compete for the same binding sites, it is plausible that elevated GATA2 may disrupt the recruitment of *HBG* repressors through displacement of GATA1 at the *HBD* promoter region.

In conclusion, our data demonstrate that selective inhibition of HDAC1/2 by ACY-957 induces *HBG* and HbF, providing a foundation for *in vivo* animal studies and further development of ACY-957 as a therapy for SCD and β-thalassemia.
